# Appendiceal Crohn's Disease Manifesting as Appendiceal Malignancy: A Case Report and Review of the Literature

**DOI:** 10.1002/kjm2.70147

**Published:** 2025-11-28

**Authors:** Ming‐Jung Meng, Tai‐Di Chen, Puo‐Hsien Le, Chia‐Jung Kuo

**Affiliations:** ^1^ Department of Gastroenterology and Hepatology Linkou Chang Gung Memorial Hospital Taoyuan Taiwan; ^2^ Department of Pathology, Chang Gung Memorial Hospital at Linkou Chang Gung University College of Medicine Taoyuan Taiwan; ^3^ College of Medicine Chang Gung University Taoyuan Taiwan; ^4^ Chang Gung Inflammatory Bowel Disease Center Taoyuan Taiwan; ^5^ Chang Gung Microbiota Therapy Center Taoyuan Taiwan; ^6^ Taiwan Association for the Study of Intestinal Diseases (TASID) Taoyuan Taiwan

Crohn's disease (CD) is a chronic inflammatory bowel disease that can affect any segment of the gastrointestinal tract, but most commonly affects the terminal ileum and colon. Appendiceal Crohn's disease (ACD) is rare, comprising 0.2%–2% of appendectomy specimens. Since its first description in 1953, ACD has been recognized as an uncommon subtype of CD that typically affects young adults [1, 2].

A 38‐year‐old previously healthy male presented with intermittent right lower quadrant pain that had persisted for 3 weeks. The patient denied systemic or other gastrointestinal symptoms. Examination revealed mild localized tenderness. A colonoscopy performed at a local clinic revealed a swollen lesion near the appendiceal orifice. Laboratory evaluations revealed leukocytosis with elevated C‐reactive protein (CRP) levels. Additional studies, including routine stool culture and serologic tests for Epstein–Barr virus (EBV), cytomegalovirus (CMV), human immunodeficiency virus (HIV), amebiasis, and *Clostridium difficile* toxin, were all negative. Abdominal computed tomography revealed an appendiceal mass with peri‐appendiceal fat stranding, cecal wall thickening, and regional lymphadenopathy (Figure 1A). A repeat colonoscopy revealed cecal inflammation without a discrete mass. The terminal ileum was intubated and showed no evidence of inflammation or malignancy (Figure 1B).

**FIGURE 1:**
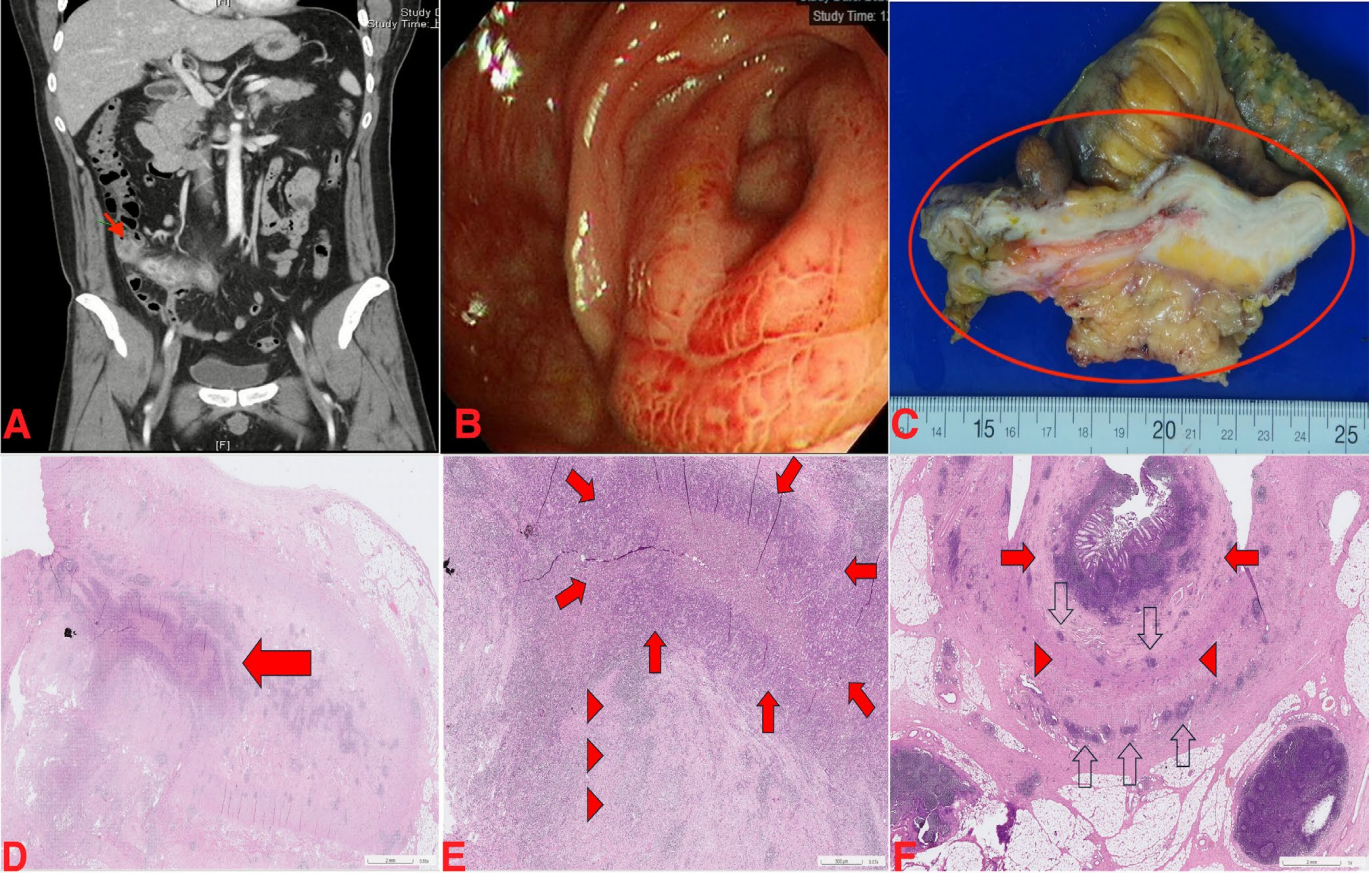
(A) Abdominal CT revealed an appendiceal mass with peri‐appendiceal fat stranding, cecal wall thickening (arrow), and regional lymphadenopathy. (B) Colonoscopy showed cecal inflammation without a discrete mass. (C) Intraoperatively, a 2 × 1 cm ulcerative mass (circle) confined to the appendix with partial ileal involvement. (D) Low power view of the appendix shows obliteration of the lumen and mucosa with marked inflammation (arrow). (E) Medium power view shows abscess formation in the appendiceal lumen with a neutrophilic cuffing (arrows) and mononuclear cell infiltrate in the muscular wall (arrow heads). (F) Another low power view shows submucosal fibrosis (arrows), muscular hypertrophy (arrow heads), and transmural lymphoid follicles both inner and outer the muscularis propria (hollow arrows).

Because of concerns about malignancy, a laparoscopic right hemicolectomy with resection of the terminal ileum and ileocolic lymph nodes was performed. Intraoperatively, a 2 × 1 cm ulcerative mass confined to the appendix, with partial involvement of the adjacent ileum and multiple enlarged ileocecal lymph nodes was observed (Figure 1C). Histopathology revealed acute and chronic transmural inflammation with fissures, lymphoid aggregates, and lymphoid follicles involving both the appendix and cecum, consistent with CD; no tuberculosis, parasitic infection or neoplasia was identified (Figure 1D–F). A diagnosis of ACD with cecal extension was established. The postoperative course was uneventful and a follow‐up colonoscopy at 6 months revealed no evidence of disease recurrence throughout the colonic lumen up to the anastomotic site.

Clinically, ACD often mimics acute appendicitis and, less frequently, appendiceal carcinoma. Radiological findings such as an appendiceal mass, fat stranding, and lymphadenopathy may be indistinguishable from malignancy [3, 4]. Colonoscopy may reveal cecal inflammation but is usually nondiagnostic. The definitive diagnosis relies on histopathology, with features such as transmural inflammation, lymphoid aggregates, and non‐caseating granulomas (if present).

Surgery is the primary treatment for this condition. Appendectomy alone is usually curative, though right hemicolectomy may be warranted when malignancy is suspected or when contiguous cecal or ileal involvement is evident. The role of long‐term surveillance remains debatable; however, periodic follow‐up is prudent to detect recurrence [5]. Our patient demonstrated no disease progression after 6 months.

## Conflicts of Interest

The authors declare no conflicts of interest.

## Data Availability

The data that support the findings of this study are available on request from the corresponding author. The data are not publicly available due to privacy or ethical restrictions.
